# Serum Concentrations of Selected Poly- and Perfluoroalkyl Substances (PFASs) in Pregnant Women and Associations with Birth Outcomes. A Cross-Sectional Study from Southern Malawi

**DOI:** 10.3390/ijerph20031689

**Published:** 2023-01-17

**Authors:** Mphatso Mwapasa, Sandra Huber, Bertha Magreta Chakhame, Alfred Maluwa, Maria Lisa Odland, Halina Röllin, Augustine Choko, Shanshan Xu, Jon Øyvind Odland

**Affiliations:** 1 Department of Public Health and Nursing, Norwegian University of Science and Technology, 7491 Trondheim, Norway; 2 School of Maternal, Neonatal and Reproductive Health, Kamuzu University of Health Sciences, Blantyre 312225, Malawi; 3 Department of Laboratory Medicine, University Hospital of North Norway, 9038 Tromsø, Norway; 4 Directorate of Research and Outreach, Malawi University of Science and Technology, Thyolo 310106, Malawi; 5 Malawi Liverpool Wellcome Trust Clinical Research Programme, Blantyre 312233, Malawi; 6 School of Health Systems and Public Health, Faculty of Health Sciences, University of Pretoria, Pretoria 0002, South Africa; 7 Centre for International Health, Department of Global Public Health and Primary Care, University of Bergen, 5009 Bergen, Norway

**Keywords:** poly- and perfluoroalkyl substances, birth outcomes, southern Malawi

## Abstract

Pervasive exposure to per-and polyfluoroalkyl substances (PFASs) shows associations with adverse pregnancy outcomes. The aim of the present study was to examine the determinants of different serum PFAS concentrations in late pregnancy and their relationship with birth outcomes in southern Malawi. The sample included 605 pregnant women with a mean age of 24.8 years and their offspring from three districts in the southern region of Malawi. Six PFAS were measured in serum from third-trimester women. The serum PFAS concentrations were assessed with head circumference, birth length, birth weight, gestational age and ponderal index. Participants living in urban areas had significantly higher serum levels of PFOA, PFNA and SumPFOS, while SumPFHxS concentrations were higher in women from rural settings. High PFOA, PFNA and SumPFHxS concentrations were generally inversely associated with head circumference. Birth length was negatively associated with PFOA and PFNA while SumPFHxS was negatively associated with birth weight. SumPFOS was inversely associated with gestational age. Urban area of residence was the strongest predictor for high PFAS concentrations in the maternal serum and was generally associated with adverse birth outcomes. The results highlight the need to investigate SumPFHxS further as it follows a pattern that is different to similar compounds and cohorts.

## 1. Introduction

Per-and polyfluoroalkyl substances (PFASs) are highly fluorinated aliphatic substances that consist of carbon (C) chains of different length with a perfluoroalkyl moiety (CnF2n+1-) [1]. These compounds are man-made synthetic chemicals that are highly resistant to biodegradation and show high affinity for bioaccumulation (due to more intake than excretion of the chemicals) and biomagnification in the environment and living organisms, including humans. Although the production and usage of perfluorooctane sulfonate (PFOS) and perfluorooctanoate (PFOA) in particular has been gradually reduced in several countries since the year 2000, human exposure continues mainly due to persistence in the environment and use. PFASs have a wide range of applications in industry as well as consumer products [2,3] and are also known to have long half-lives. For instance, it is estimated that the arithmetic and geometric mean half-lives of serum elimination in human beings for PFOS, perfluorohexane sulfonate (PFHxS) and PFOA are as follows: 5.4 years and 4.8 years for PFOS; 8.5 years and 7.3 years for PFHxS; and 3.8 years and 3.5 years for PFOA, respectively [4]. Furthermore, PFASs are able to undergo atmospheric and marine long-range transport and are hence found in remote areas such as the Antarctic and Arctic, far away from their areas of production and use [5,6,7].

Globally, there are growing concerns about the links between exposure to PFAS compounds and adverse health effects [8]. A wide range of adverse health effects associated with different single PFASs but also sum concentration of PFASs were observed in previously published studies [9]. The health effects of concern related to PFASs include altered metabolism and fertility [10], increased risk of being overweight or obese [11] and reduced ability of the immune system to fight infections [12]. New research indicates that PFASs have the ability to transfer from mother to child through the placenta. In this regard, there is growing concern over possible adverse impacts on development and health later in life due to early exposure to these chemicals [13]. Of particular concern are both short-term and long-term subtle effects that might influence reproductive health, pregnancy outcomes, reduce defense against diseases and increase the risk of cancer [14]. 

A number of studies have suggested an association between higher concentrations of some PFAS and low birth weight [15,16,17,18,19], preterm birth [17,18], birth length [20] and gestational age [21]. Most of the monitoring and research on PFASs has been conducted in developed countries, especially in Europe and North America. Only a few studies were published from developing countries and countries situated in the southern hemisphere. Biomonitoring data from the northern hemisphere may not be applicable to the southern hemisphere. In this regard, results and findings from Malawi and other countries situated in the southern hemisphere are of particular importance and needed in order to evaluate the current exposure situation to PFASs in these regions together with associations related to health outcomes in general. Furthermore, although the WHO developed PFOA and PFOS guidelines for drinking water standards, there are recommendations from public health advocates, scientists and organizations for a revision or withdrawal of the guidelines. The call for the revision or withdrawal of the guidelines follows an argument that they are neither health protective nor based on the best available scientific evidence, and hence, they are more likely to promote global health inequities [22]. In this regard, data from this study may provide a foundation to be used in the process of revision of the above-stated guidelines. The present study on pregnant women from three different locations in Malawi was conducted with the aim to examine the determinants of different serum PFAS concentrations in late pregnancy and investigate their relationship with birth outcomes. 

## 2. Materials and Methods

### 2.1. Study Design and Study Population

This is a cross-sectional study of delivering women giving birth and their offspring. The study was conducted in the southern region of Malawi, in antenatal clinics/wards and labor wards of three government health facilities. The health facilities that were randomly selected for the study include Ndirande Health Centre, Chiradzulu District Hospital (CDH) and Thyolo District Hospital (TDH). Ndirande Health Centre is located in Blantyre, which is the commercial city of Malawi, while Chiradzulu and Thyolo district hospitals are located in Thyolo and Chiradzulu districts, respectively. All study sites are located in the southern region of Malawi. Ndirande Health Centre is situated in an urban setting while the other district hospitals represent the rural setting. 

### 2.2. Data Collection and Management

**Figure 1:**
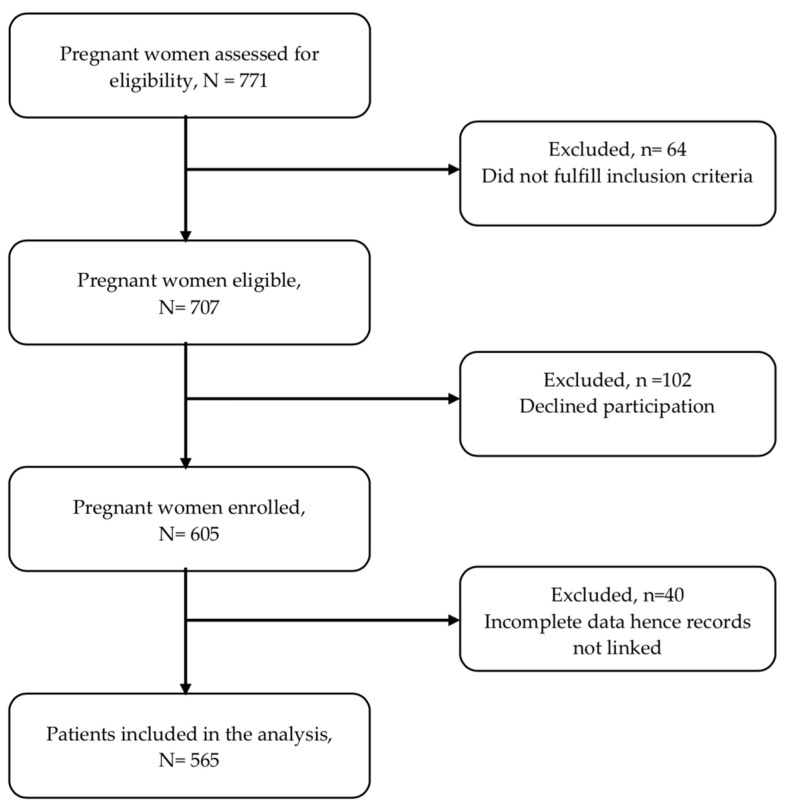
Flow chart of included and excluded participants.

Study participants were recruited between August 2020 and July 2021 using data collection tools that were pretested in a pilot survey conducted soon before the main survey. Personal characteristics, socioeconomic status, lifestyle, infant information and environmental characteristics were collected through a questionnaire administered by a trained research nurse. A total of 605 women and neonate pairs were recruited into the study. However, 40 were excluded from the serum PFAS analysis due to a lack of biological samples, yielding a final study population of 565. Figure 1 gives details on the numbers in the recruitment process.

### 2.3. Serum Blood Sample Collection and Preliminary Analysis

Blood samples were collected from the mothers at an optimal time before delivery (36 ± 12 h prior to delivery). Methods for collection and transportation of whole blood and serum samples were adapted from CTQ Laboratory guidelines, Quebec, Canada [23]. In brief, venous blood was collected from the mother using a 5 mL red-top BD Vacutainers^®^ (REF # 367614). The red-top vacutainer containing the whole blood was then left to stand at room temperature for about 60 min for complete clot formation before centrifugation at ≤1200× *g* (3276 rotations per minute) for 10 min at room temperature (18–25 °C). After centrifugation, the serum was transferred to two glass tubes with green lids (27138 Sigma Aldrich, St. Louis, MO, USA) using a disposable glass pipette. Transfer of serum to the green-lid tubes was performed with caution to avoid pipetting the red cells along with the serum. All collected biological samples were stored in a freezer at a temperature between −35 °C and −20 °C before being shipped to University Hospital of North Norway (UNN), Department of Laboratory Medicine, for analysis.

### 2.4. Serum Sample Analysis

Sample preparation, instrumental analysis, quantification and quality controls have been described in detail elsewhere [24]. Briefly, extracts were prepared by an automated liquid handler Tecan Freedom Evo 200 (Männedorf, Switzerland). Perfluorobutane sulfonate (PFBS), perfluoropentane sulfonate (PFPS), perfluorohexane sulfonate (PFHxS), perfluoroheptane sulfonate (PFHpS), PFOS, perfluorononane sulfonate (PFNS), perfluorodecane sulfonate (PFDS), perfluorododecane sulfonate (PFDoDS), perfluorooctane sulfonamide (PFOSA), perfluorohexanoate (PFHxA), perfluoroheptanoate (PFHpA), PFOA, perfluorononanoate (PFNA), perfluorodecanoate (PFDA), perfluoroundecanoate (PFUnDA), perfluorododecanoate (PFDoDA), perfluorotridecanoate (PFTrDA) and perfluorotetradecanoate (PFTeDA) were analyzed by ultrahigh pressure liquid chromatography tandem-quadrupole mass-spectrometry (UHPLC-MS/MS). Sum of branched and linear species (Σ) was quantified for PFHxS, PFHpS, PFOS, PFHxS, PFHpS, PFNS, PFDS and PFOSA. Analyses were performed with a Waters Acquity UPLC system (Waters, Milford, MA, USA) consisting of a binary solvent manager, an autosampler and a column manager coupled to a Xevo TQ-S mass spectrometer (Waters, Milford, MA, USA) through an atmospheric pressure electrospray interface. Separation of the target analytes was achieved on an Acquity UPLC HSS T3 column (2.1 × 100 mm, 1.8 µm) (Waters, Milford, MA, USA) by using a programmed flow and solvent gradient of 2 mM NH_4_OAc in MilliQ-water and 2 mM NH_4_OAc in methanol as mobile phase. Quantification was conducted by applying the Masslynx and Targetlynx software (Version 4.1, Waters, Milford, MA, USA) and achieved by the internal standard method with isotope-labelled PFASs. Method quantification level (MQL) was defined as ten times a signal-to-noise ratio or three times the limit of detection (LOD). LODs (minimum limit of detection) were set as concentrations calculated by the Targetlynx software for each individual sample (LODi) and each individual analyte with a signal-to-noise ratio of 3 divided by the related sample amount. Where blank contamination was detected (background contribution during sample preparation), a blank subtraction was performed batch wise by calculating an average of the blanks added to three times their standard deviation. An eight-point calibration curve with concentrations ranging from 0.01 pg/µL to 10 pg/µL was applied for quantification. 

#### Quality Controls

Accuracy and precision of the method was described in detail in the associated method article [24]. For quality assurance, 4 blank samples, 4 SRM 1957 and SRM 1958 (NIST, Gaithersburg, MD, USA) samples and 3 bovine serum samples (Sigma Aldrich, Steinheim, Germany) were analyzed within each batch of 96 samples. Differences from the assigned mean reference concentrations were from 3 to 13% for SRM 1957 and 3 to 9% for SRM 1958 during the Malawi study. Additionally, our laboratory participates successfully in the international quality control program: the Arctic Monitoring and Assessment (AMAP) Ring Test for Persistent Organic Pollutants in Human Serum (organized by the Laboratoire de toxicologie, Institut national de santé publique du Quebec, Canada). Solvent injections were performed regularly during instrumental analysis in order to monitor instrument background and carryover effects.

### 2.5. Measurement of Birth Outcomes

Birth outcome variables measured in this study were gestational age (weeks), birth weight (kilograms), birth length (cm), head circumference (cm) and ponderal index (kg/m^3^). Ponderal index was calculated using the following formula: ponderal index = weight(kg)/height^3^ (m^3^).

### 2.6. Statistical Analysis

Statistical analyses were carried out using the Stata for Mac (SE standard version 17; College Station, TX, USA). A total of 24 compounds were analyzed, but most of the compounds were under the limit of detection. However, 6 out of the 24 PFASs analyzed compounds—namely PFOA, PFNA, PFDA, PFUDA, SumPFHxS and SumPFOS—had detection limits of over 60% and hence were used for the statistical analysis. Data were given as arithmetic means, standard deviation (SD), median, and minimum and maximum or proportion (%) for describing sociodemographic characteristics of the study population. A significance level of *p* < 0.05 (two-tailed) was set for all analyses. 

PFAS concentrations were log-transformed before assessing linear associations due to non-normal distribution of the concentrations among participants.

### 2.7. Ethical Considerations

The study was carried out following ethical rules and guidelines. Ethical clearance was obtained from the College of Medicine Research and Ethics Committee (COMREC)- Malawi (P.11/18/2546) and REK-Norway (#355656 2020). Permission to conduct the study at the selected sites was sought from Blantyre (for Ndirande Health Centre), Chiradzulu (for CDH) and Thyolo (for TDH) district health offices. Participation in the study was voluntary, based on signed written consent from the mother. Confidentiality was maintained by assigning pseudo-anonymized identification numbers to all study participants. The sampling of blood and extraction of information did not interfere with the health service delivery process and took place either before or after delivery, depending on the individual circumstances of each study participant.

## 3. Results

### 3.1. Maternal Sociodemographic Data and Neonate Birth Characteristics

The selected maternal sociodemographic characteristics of the n = 605 individuals are presented in Table 1. 

Participants ages ranged from 16 to 45 years, with a mean (SD) age of 24.8 (6.2) years. Out of the 605 participants recruited, 308 pregnant women were recruited from urban (Ndirande Health Centre) and 297 from rural (Chiradzulu and Thyolo district hospitals) settings. In this regard, mean age of the pregnant women recruited from the urban setting was almost 2 years older than those from rural areas, with a mean of 25.6 (SD = 6.7) and 23.9 (SD = 5.7) years of age, respectively. Similarly, the mean age of spouses from the urban setting was 30.7 (SD = 6.4) versus 28.1 (SD = 7.8) for rural. 

The data showed a significant difference in gravidity, parity and educational levels between the two groups. In this regard, nulliparity was significantly high in rural areas. Para 1 and multiparty were statistically high in urban areas as compared to rural.

Over half (69.7%) of the women from the rural areas either did not attend any formal school or were educated up to primary level only as compared to only 38.6% from the urban area. Furthermore, a vast proportion (61.4%) of those recruited from the urban area attained education up to secondary or tertiary level, while only 30.3% from the rural area attained such levels. A high percentage of women (96.8%) residing in the urban areas used tap water as their source of drinking water in comparison to only 8.1% from the rural locations. Shallow wells and boreholes were the most common (44.1% and 45.5%, respectively) sources of drinking water for the rural study participants. 

A total of 572 neonates were recruited in this study. Out of this, 296 were boys (51.8%). The mean birth weight, length and head circumference were 3.09 kg, 45.28 cm and 33.15 cm, respectively, in the overall sample. Detailed information about neonates according to area of residence (urban versus rural) is also outlined in Table 1.

### 3.2. Maternal PFASs Serum Concentrations 

Six out of the twenty-four PFASs analyzed compounds—PFOA, PFNA, PFDA, PFUDA, SumPFHxS and SumPFOS—had detection limits of over 60% and hence were used for the statistical analyses. Table 2 outlines the serum concentration in ng/mL of the above listed 6 PFASs. The highest PFAS median concentrations found in this study were 3.09 ng/mL and 0.533 ng/mL for SumPFHxS and SumPFOS, with detection rates of 99.8% and 99.5%, respectively. The lowest median concentration levels of all the PFASs assessed was 0.018 ng/mL for PFUDA. The measured maternal serum PFASs in descending order of median concentration were SumPFHxS > SumPFOS > PFOA> PFDA> PFNA > PFUDA.

### 3.3. PFASs in Serum and Maternal Characteristics 

Multiple linear regression analysis of maternal sociodemographic/lifestyle characteristics versus the concentrations of different PFASs provided a relatively comprehensive description of the main maternal risk factors related to serum PFAS levels (Table 3).

Adjusted for maternal age, parity, maternal educational level, area of residence (urban vs. rural) and source of drinking water, living in the rural setting was associated with decreased maternal PFOA (β = −0.581; 95% CI: −0.957 to −0.204; *p* = 0.003), PFUDA (β = −0.412; 95% CI −0.812 to −0.013; *p* = 0.043) and SumPFOS (β = −1.535; 95% CI: −2.943 to −0.127; *p* = 0.003) serum concentrations. Conversely, high concentrations of SumPFHxS were associated with living in rural areas (β = 1.715; 95% CI: 0.067 to 3.363; *p* = 0.041). However, there was no statistically significant inverse association between serum PFNA and PFDA concentrations and area of residence.

### 3.4. PFAS Concentrations and Birth Outcomes 

Multiple linear regression analysis of birth outcomes and maternal serum PFAS concentrations shows the possible associations between different maternal serum PFAS concentrations and birth weight, head circumference, birth length, gestational age and ponderal index. These results were examined by multiple regression analysis while adjusting for maternal age, area of residence (urban vs. rural), maternal educational level, parity and source of drinking water (Table 4). A statistically significant inverse association was observed between the natural log-transformed maternal serum of PFOA concentrations (In_PFOA) and head circumference (β = −0.056; 95% CI: −0.109 to −0.002; *p* = 0.043). Similarly, a negative association was also observed between In_PFOA and birth length (β = −0.049; 95% CI: −0.077 to −0.022; *p* < 0.001). Conversely, a positive association was observed between In_PFOA and ponderal index (β = 0.136; 95% CI: 0.040 to 0.233; *p* = 0.005). No associations were observed between In_PFOA and birth weight or gestational age in both models.

There was a statistically significant negative relationship between natural log-transformed maternal serum PFNA concentrations (In_PFNA) and head circumference (β = −0.080; 95% CI: −0.125 to −0.035; *p* = 0.001). Similarly, negative associations were also observed between In_PFNA and birth length (β = −0.033; 95 CI: −0.057 to −0.010; *p* = 0.005) and gestational age (β = −0.083; 95% CI: −0.141 to −0.023; *p* = 0.005). However, there was no statistically significant association between In_PFNA and birth weight and ponderal index. Furthermore, a statistically significant negative association was observed between natural log of maternal serum SumPFHxS (In_SumPFHxS) concentrations and birth weight (β = −0.189; 95% CI: −0.371 to −0.006; *p* = 0.043). Similarly, an inverse statistically significant association was observed between In_SumPFHxS and ponderal index (β = −0.090; 95% CI: −0.175 to −0.005; *p* = 0.037).

Another statistically significant negative association was observed between the natural log of SumPFOS (In_SumPFOS) serum concentrations and birth weight (β = −0.261; 95% CI: −0.457 to −0.064; *p* = 0.009). An inverse association was also observed between In_SumPFOS and gestational age (β = −0.119; 95% CI: −0.183 to −0.055; *p* < 0.001). Conversely, statistically significant positive associations were observed between In_PFOA and ponderal index (β = 0.136; 95% CI: 0.040 to 0.233; *p* = 0.005). A similar trend was also detected between In_SumPFHxS and head circumference (β = 0.048; 95% CI: 0.001 to 0.095; *p* = 0.045).

## 4. Discussion

Area of residence was the main determinant of maternal serum PFAS, with higher concentrations registered from urban settings than from rural areas. Increased concentrations of some maternal serum PFASs (i.e., PFOA, PFNA, SumPFHxS and SumPFOS) were mostly inversely associated with some, but not all, birth outcomes. There were very few notable positive associations between PFASs and birth outcomes as follows: SumPFHxS with head circumference (cm) and PFOA with ponderal index.

Maternal serum PFOA, PFDA and PFUDA concentrations observed in this study were lower compared to other studies from Sweden, Norway, Canada, Spain, Russia, Uzbekistan and Denmark [25,26,27,28,29,30,31]. In addition, PFOA and PFNA concentrations were also lower than those observed in a South African study [32]. In this regard, the results from the present study are suggestive of the effects of year of sampling and different lifestyles between Malawi and the other areas that were used for comparison. For instance, most women recruited in this study reported either local production or local markets (99.3%) as their main source of food for consumption, with only 0.7% of women reporting consumption of supermarket and imported foods. The above result adds weight to suggestions from other studies conducted in North America and Europe which concluded that imported food, fast food, and preprepared food packed in food packaging material are the main source of PFAS exposure. However, the maternal serum PFNA concentrations observed in our study were close to the results found in the Estudio del Medio Ambiente y la Salud Reproductiva (EMASAR) study that compared PFAS concentrations in maternal serum between two different regions in Argentina, Ushuaia and Salta [33]. Nevertheless, they were slightly low as compared to the results from a Norwegian study conducted by Berg et al. [26]. 

The median for maternal SumPFOS concentrations observed in the present study (0.533 ng/mL) was slightly lower that the levels observed in Ushuaia and Salta (0.84 and 0.70 ng/mL, respectively) regions in Argentina [33]. However, the levels detected are very low as compared to results from a study conducted by Starling et al. (12.9 ng/mL) in Norway [29]. The underlying reasons for such a difference could be due to the different lifestyles between the areas. As already discussed above, our sample constituted women that predominantly use local production and local markets as their main source of food as compared to both Argentina and Norway, hence the lower probability of exposure to SumPFOS. In contrast, the maternal serum SumPFHxS concentrations observed in our study were very high as compared to other studies conducted elsewhere. For instance, the median SumPFHxS in our study was 3.09 ng/mL against 0.18 ng/mL and 0.22 ng/mL for Ushuaia and Salta, respectively, in Argentina.

Area of residence was one of the main determinants for the blood serum PFAS concentrations, with higher blood serum PFOA and PFNA concentrations detected in those living in urban areas than those living in rural areas. This difference could be explained by differences in socioeconomic conditions and lifestyles, including dietary habits. PFASs are used in many industrial and consumer applications such as textile impregnation, paper production, fire-fighting foam, lubricants production and electroplating [2]. These compounds are more likely to be produced and used in urban settings. In this regard, the odds of exposure to these compounds to mothers residing in the urban areas is expected to be high as compared to rural settings.

Drinking water is known to be one of the main sources of PFASs exposure for humans [34,35,36,37]. In this regard, the source of drinking water could also be the reason for elevated serum PFAS concentrations in urban study participants. The majority of urban study participants recruited use tap water (surface water) as a source of drinking water. Boreholes (ground water) are the main source of drinking water for their counterparts from the rural setting. However, there is a need for further studies to assess the level of PFASs between tap and borehole water to evaluate this hypothesis.

Various research studies have shown that both PFOA and PFOS can cross the placental barrier [38,39,40,41,42,43,44] and affect neonate birth weight, birth length and gestational age. In this regard, high PFAS concentration in the placenta may presumably restrict fetal growth. The results of the present study did not show a clear association between PFOA and birth weight (*p* > 0.05). These results are consistent with the Brazilian Ribeirão Preto (BRISA) study and a systematic review that included results from nine different studies that found no significant association between PFOA and birth weight [45,46]. In contrast, we observed an inverse association between SumPFOS and birth weight (*p* = 0.009). Similarly, the meta-analysis of 23 papers conducted by Yang et al. [47] also found a negative association between PFOS and birth weight. SumPFOS was also indicated to be inversely associated with gestational age (*p* < 0.001). However, the results showed a statistically significant inverse association between maternal serum PFOA concentrations and head circumference. Furthermore, there was also an inverse association between PFOA concentrations and length at birth. 

Increased maternal serum PFNA concentrations in the present study were statistically associated with low birth weight. This finding was similar to the study conducted in Spain by Manzano-Salgado et al. [19]. However, our findings become statistically nonsignificant after adding source of drinking water to the line of covariates. This could be due to confounding of the association by source of drinking water.

Maternal serum SumPFHxS concentrations showed a statistically significant association with low level of education. This association may be reflective of behavior and lifestyle factors associated with socioeconomic status. Unexpectedly, a statistically significant positive association was observed between maternal SumPFHxS concentrations and head circumference. This is in contrast to a study by Xiao et al. [48] that observed an inverse association between PFHxS and head circumference. Furthermore, another study conducted in the Spanish cohort did not find any association between the two variables [19]. The differences in the results between the current study and the other two could be explained by different sample sizes and the methodologies that were used. In this regard, there is a need for more research assessing the relationship between individual PFASs and head circumference. No statistically significant associations were observed between parity and each of the PFASs assessed in the present study. These findings are in contrast to other studies that observed significantly higher PFOA concentrations in nulliparous compared to multiparous women [49,50].

Our study has several important strengths. To our knowledge, this is the first study assessing PFAS concentrations in delivering women from Malawi. In this regard, our study provides highly valuable benchmark information on PFAS exposure status of delivering women from Malawi. Furthermore, our study contributes to a body of knowledge on the rather underrepresented research area on PFAS from the African continent. Different socioeconomic status and lifestyle sites were included in the present study, hence providing a representative sample for all settings. Furthermore, we measured a panel of 24 PFASs, including PFDA, PFNA and PFHxS, which have not been as extensively studied as PFOA and PFOS. We also acknowledge several limitations of our study. The number of patients assessed for eligibility matched the sample size calculation. However, some participants refused to have their blood sample collected, which reduced the total number of data analyzed in the final data set. However, we assumed that this would not have a significant effect on the power of the current study.

## 5. Conclusions

The present study explores and presents PFAS concentrations among delivering women in Malawi. Area of residence was the predictor for high concentrations of PFASs detected in serum of women from urban settings. Maternal serum PFAS concentrations were associated with some but not all birth outcomes. PFAS concentrations assessed in the present study, except SumPFHxS, are lower as compared to other parts of the world. Follow-up studies are needed to evaluate the association between the source of drinking water and maternal serum PFAS concentrations. There is a need to conduct further investigations on PFHxS as it follows a totally different pattern compared to similar compounds and many other cohorts.

## Figures and Tables

**Table 1:** Sociodemographic characteristics of the mothers and neonates.

		Place of Residence			
Variable	Characteristic	Total	Urban	Rural	*p*-Value
Total participants (n)		605	308	297	
Age (years)	Mean (SD)	24.80(6.22)	25.63 (5.65)	23.93 (6.66)	<0.001
Gravidity (%)	1	220 (36.4)	78 (25.4)	142 (47.7)	<0.001
	2	157(26.0)	106 (34.5)	51 (17.1)	
	3	117 (19.3)	73 (23.8)	44 (14.8)	
	4	111 (18.4)	50 (16.3)	61 (20.5)	
Parity (%)	0	207 (34.5)	61 (20.2)	146 (49.0)	<0.001
	1	137 (22.8)	86 (28.5)	51 (17.1)	
	2	256 (42.7)	155 (51.3)	101 (33.9)	
Education level of mother (%)	None/primary	325 (53.9)	118 (38.6)	207 (69.7)	<0.001
	Secondary/tertiary	278 (46.1)	188 (61.4)	90 (30.3)	
Marital status (%)	Married	544 (89.8)	283 (91.9)	261 (87.6)	0.081
	Single	62 (10.2)	25 (8.1)	37 (12.4)	
Breast feeding (%)	No	246 (40.9)	98 (32.0)	148 (50.2)	<0.001
	Yes	355 (59.1)	208 (68.0)	147 (49.8)	
Source of drinking water, count (%)	Tap	322 (53.5)	298 (96.8)	24 (8.2)	<0.001
	Lake/shallow well	137 (22.8)	2 (0.7)	135 (45.9)	
	Borehole	143 (23.8)	8 (2.6)	135 (45.9)	
Use of pesticides at home (%)	Do not use pesticides	480 (79.5)	288 (93.5)	192 (64.9)	<0.001
	Pesticides	124 (20.5)	20 (6.5)	104 (35.1)	
Fishing (%)	Do not Fish	596 (98.5)	308 (100.0)	288 (97.0)	0.002
	Fish	9 (1.5)	0 (0.0)	9 (3.0)	
Gestational age (weeks)	Mean (SD)	37.59 (1.53)	37.47 (1.43)	37.71 (1.62)	0.075
Birth weight (kg)	Mean (SD)	3.09 (0.46)	3.18 (0.45)	3.00 (0.46)	<0.001
Birth length (cm)	Mean (SD)	45.19 (3.46)	45.95 (4.08)	44.53 (2.66)	<0.001
Head circumference (cm)	Mean (SD)	33.17 (1.83)	33.14 (1.94)	33.19 (1.72)	0.753
Ponderal index (kg/m^3^)	Mean (SD)	3.43 (0.97)	3.44 (1.23)	3.43 (0.66)	0.874

SD: standard deviation of mean.

**Table 2:** Maternal serum concentrations (ng/mL) of PFASs.

Maternal Serum Concentrations (ng/mL) of PFASs (n = 565)			
PFASs	% > LOD	Mean (SD) ^a^	Median (Min–Max)
PFOA	95.2	0.18 (0.31)	0.12 (0.002–2.66)
PFNA	96.3	0.05 (0.09)	0.04 (0.001–1.94)
PFDA	93.8	0.07 (0.05)	0.06 (0.002–0.490)
PFUDA	60.2	0.02 (0.02)	0.02 (0.002–0.160)
SumPFHxS	99.8	4.68 (4.59)	3.09 (0.001–28.3)
SumPFOS	99.5	1.40 (4.32)	0.53 (0.002–56.7)

^a^ Arithmetic mean with standard deviation (SD). The limit of detection (LOD) > 60% of the samples.

**Table 3:** Multivariable linear regression of PFAS concentrations in blood serum and maternal characteristics.

	Maternal Characteristics		Maternal Serum PFAS Concentrations ^a^		
			n	β (95% CI)	*p*-Value
PFOA	Maternal age (years)		537	−0.005 (−0.025 to −0.015)	0.649
	Parity		537		
		Para 0		Reference category	
		Para 1		0.278 (0.026 to 0.531)	0.031
		Multiparity		0.105 (−0.190 to 0.401)	0.484
	Education of mothers		537		
		None/primary		Reference category	
		Secondary/tertiary		0.093 (−0.091 to 0.277)	0.32
	Area of residence		537		
		Urban		Reference category	
		Rural		−0.581 (−0.957 to −0.204)	0.003
	Source of drinking water		537		
		Tap		Reference category	
		Lake/shallow well		0.645 (0.252 to 1.039)	0.001
		Borehole		−0.124 (−0.503 to 0.225)	0.521
PFNA	Maternal age (years)		537	0.005 (−0.012 to 0.022)	0.562
	Parity		537		
		Para 0		Reference category	
		Para 1		0.117 (−0.098 to 0.331)	0.286
		Multiparity		0.020 (−0.231 to 0.271)	0.874
	Education of mothers		537		
		None/primary		Reference category	
		Secondary/tertiary		0.020 (−0.136 to 0.176)	0.801
	Area of residence		537		
		Urban		Reference category	
		Rural		−0.316 (−0.636 to 0.003)	0.052
	Source of drinking water		537		
		Tap		Reference category	
		Lake/shallow well		0.311 (−0.023 to 0.645)	0.068
		Borehole		0.022 (−0.300 to 0.343)	0.895
PFDA	Maternal age (years)		537	−0.004 (−0.022 to 0.013)	0.65
	Parity		537		
		Para 0		Reference category	
		Para 1		0.086 (−0.135 to 0.306)	0.445
		Multiparity		0.099 (−0.160 to 0.358)	0.452
	Education of mothers		537		
		None/primary		Reference category	
		Secondary/tertiary		0.140 (−0.021 to 0.301)	0.089
	Area of residence		537		
		Urban		Reference category	
		Rural		−0.170 (−0.500 to 0.159)	0.311
	Source of drinking water		537		
		Tap		Reference category	
		Lake/shallow well		0.058 (−0.286 to 0.402)	0.741
		Borehole		−0.155 (−0.487 to 0.177)	0.359
PFUDA	Maternal age (years)		537	0.007 (−0.014 to 0.028)	0.531
	Parity		537		
		Para 0		Reference category	
		Para 1		0.022 (−0.246 to 0.290)	0.873
		Multiparity		−0.036 (−0.350 to 0.278)	0.822
	Education of mothers		537		
		None/primary		Reference category	
		Secondary/tertiary		0.128 (−0.067 to 0.323)	0.199
	Area of residence		537		
		Urban		Reference category	
		Rural		−0.412 (−0.812 to −0.013)	0.043
	Source of drinking water		537		
		Tap		Reference category	
		Lake/shallow well		−0.068 (−0.485 to 0.350)	0.749
		Borehole		−0.024 (−0.426 to 0.379)	0.908
SumPFHxS	Maternal age (years)		537	−0.057 (−0.145 to 0.031)	0.202
	Parity		537		
		Para 0		Reference category	
		Para 1		−0.127 (−1.233 to 0.979)	0.822
		Multiparity		0.386 (−0.908 to 1.680)	0.558
	Education of mothers		537		
		None/primary		Reference category	
		Secondary/tertiary		−1.073 (−1.878 to −0.268)	0.009
	Area of residence		537		
		Urban		Reference category	
		Rural		1.715 (0.067 to 3.363)	0.041
	Source of drinking water		537		
		Tap		Reference category	
		Lake/shallow well		−0.577 (−2.299 to 1.145)	0.511
		Borehole		1.077 (−0.583 to 2.737)	0.203
SumPFOS	Maternal age (years)		537	0.037 (−0.038 to 0.112)	0.331
	Parity		537		
		Para 0		Reference category	
		Para 1		−0.701 (−1.646 to 0.243)	0.145
		Multiparity		−0.866 (−1.972 to 0.240)	0.125
	Education of mothers		537		
		None/primary		Reference category	
		Secondary/tertiary		−0.441 (−1.129 to 0.246)	0.209
	Area of residence		537		
		Urban		Reference category	
		Rural		−1.535 (−2.943 to −0.127)	0.033
	Source of drinking water		537		
		Tap		Reference category	
		Lake/shallow well		−0.145 (−1.616 to 1.326)	0.847
		Borehole		−0.348 (−1.767 to 0.071)	0.63

^a^ Maternal blood PFAS concentrations were natural log-transformed. All association between PFAS concentrations in serum and maternal characteristics were adjusted for maternal age, parity, maternal educational level, area of residence (urban vs. rural) and source of drinking water.

**Table 4:** Multivariable analysis results of linear regression analysis measuring effects of maternal serum PFAS concentrations on birth outcomes.

	Outcomes		Maternal Serum PFAS Concentrations ^a^	
		n	β (95% CI)	*p*-Value
PFOA	Head Circumference (cm)	478	−0.056 (−0.109 to −0.002)	0.043
	Birth Length(cm)	478	−0.049 (−0.077 to −0.022)	<0.001
	Birth Weight (kg)	508	−0.067 (−0.272 to 0.138)	0.523
	Gestational Age (weeks)	480	−0.036 (−0.103 to 0.032)	0.298
	Ponderal Index (kg/m^3^)	477	0.136 (0.040 to 0.233)	0.005
PFNA	Head Circumference (cm)	478	−0.080 (−0.125 to −0.035)	0.001
	Birth Length(cm)	478	−0.033 (−0.057 to −0.010)	0.005
	Birth Weight (kg)	508	−0.171 (−0.346 to 0.003)	0.054
	Gestational Age (weeks)	480	−0.083 (−0.141 to −0.023)	0.005
	Ponderal Index (kg/m^3^)	477	0.035 (−0.050 to 0.116)	0.404
PFDA	Head Circumference (cm)	478	0.012 (−0.033 to 0.057)	0.597
	Birth Length(cm)	478	0.012 (−0.012 to 0.035)	0.33
	Birth Weight (kg)	508	0.038 (−0.139 to 0.25)	0.673
	Gestational Age (weeks)	480	−0.016 (−0.075 to 0.043)	0.589
	Ponderal Index (kg/m^3^)	489	−0.39 (−0.119 to 0.041)	0.340
PFUDA	Head Circumference (cm)	478	0.019 (−0.036 to 0.075)	0.494
	Birth Length(cm)	478	0.001 (−0.027 to 0.030)	0.924
	Birth Weight (kg)	508	0.052 (−0.165 to 0.269)	0.636
	Gestational Age (weeks)	480	−0.0122 (−0.083 to 0.059)	0.735
	Ponderal Index (kg/m^3^)	477	−0.026 (−0.125 to 0.074)	0.608
SumPFHxS	Head Circumference (cm)	478	0.048 (0.001 to 0.095)	0.045
	Birth Length(cm)	478	0.014 (−0.011 to 0.038)	0.265
	Birth Weight (kg)	508	−0.189 (−0.371 to −0.006)	0.043
	Gestational Age (weeks)	480	−0.160 (−0.076 to 0.044)	0.600
	Ponderal Index (kg/m^3^)	477	−0.090 (−0.175 to −0.005)	0.037
SumPFOS	Head Circumference (cm)	478	−0.036 (−0.087 to 0.014)	0.153
	Birth Length(cm)	478	−0.012 (−0.038 to 0.014)	0.348
	Birth Weight (kg)	508	−0.261 (−0.457 to −0.064)	0.009
	Gestational Age (weeks)	490	−0.119 (−0.183 to −0.055)	<0.001
	Ponderal Index (kg/m^3^)	477	−0.245 (−0.115 to 0.065)	0.591

^a^ Maternal blood PFAS concentrations were natural log-transformed. All associations between birth outcomes and PFAS levels in model A were adjusted for maternal age, area of residence (urban vs. rural), maternal educational level, parity and source of drinking water.

## Data Availability

Data will be made available upon reasonable request to the corresponding author.
